# The Open-Air Treatment of Consumption

**Published:** 1899-07-22

**Authors:** 


### THE OPEN-AIR TREATMENT OF CONSUMPTION.

By the erection of sanatoria in various parts of the country the open-air treatment is being gradually brought within the reach of a considerable number of those who suffer from consumption, and by the experience gained at these various establishments a good deal is being learned in regard to the essentials to success. Among other things it is being seen that, great as is the part played by open air, this is not everything. A consumptive cabman undoubtedly gets on better—that is, he deteriorates more slowly—than a consumptive tailor. Still, notwithstanding his 12 or 14 hours a day in the open, he does deteriorate, and very often his disease makes fatal progress, while the patient undergoing a treatment which involves perhaps only from four to six hours a day out of doors makes steady improvement, and is restored to a large degree of health, or, in popular parlance, is cured. To gain this end something more than merely dumping a patient in the open is evidently required. If we refer to the series of articles which have appeared in this month's *Practitioner* on "The Open-air Sanatorium Treatment of Phthisis in the British Islands," to an article by Dr. Pruen in the *West London Medical Journal*, and to many other writings which have of late appeared on the subject, it becomes clear how much besides mere open air is required if a consumptive patient is to have the best opportunity of recovery. As is forcibly put by Dr Pruen, fresh air merely prevents the acting of more poison on the tissues, it does not repair the damage caused by that which has already attacked them. The application of fresh air is an instance of preventive rather than curative medicine. It is the cutting of the rope which is rapidly strangling the patient, but is not in itself sufficient; and, although the very first essential in dealing with phthisis is that fresh air and nothing but fresh air shall be breathed, at all times both day and night, and though no assistance can possibly avail until this condition has been absolutely complied with, yet further steps are generally necessary to success. In the hygienic treatment of phthisis the following points seem the most important:—

**1. Purity of Air.**—This can be secured in by far the easiest manner and the highest perfection by living out of doors in the country. The difficulty in all cases arises chiefly in regard to the indoor arrangements which always have to be made for meals, for dressing, and for sleeping; and in proportion as these are efficient for securing absolute cleanliness and a free supply of fresh air, will be the success of the treatment.

It is in this that residence in a sanatorium generally compares so favourably with treatment at home. It is easy enough in a garden, and, as has been shown by Dr. Burton-Fanning, it is possible even in a yard in the centre of a town to make arrangements for open-air treatment, so far as the outdoor part of the business is concerned; the difficulty in an ordinary house, inhabited by ordinary people going about their ordinary business, is to make arrangements for securing pure air during the hours spent indoors. It can be done, but it is troublesome.

**2. A Very Full Diet.**—There is some difference of opinion as to the necessity of what has been termed "forced feeding." According to Dr. Philip, of Edinburgh, no question of compulsion, or even coaxing, need arise. The appetite returns, and there is a craving for food which is not easily satisfied, so that the term "forced feeding" is inapplicable. But whether "forced" or not, there is a general consensus that it is well that a very large amount of food should be taken; and at Nordrach, which is the model on which so many other institutions are founded, the taking of a very considerable amount of food is looked upon as a *sine quâ non.* It may be as well to point out, however, that along with this so-called "forced feeding" it is one of the rules at Nordrach that no more than three meals shall be taken. The constant irritation and disturbance to which the stomachs of some phthisical patients are subjected is, no doubt, a leading cause of much of their dyspepsia.

**3. A General Hardening of the System.**—In some places this is attained by various hydrotherapeutic proceedings, but more generally by graduated exercise and by the patients accustoming themselves to exposure to all sorts of weather. The outcome of this and the open air life is that "colds" are practically unknown, and it is difficult not to believe that one of the most important factors in the benefit derived from the open-air treatment is the freedom which the patients obtain from catarrhal attacks.

**4. Constant Medical Supervision.**—No one can read the papers to which reference has been made without seeing how important is the constant medical supervision to which patients in some of the sanatoria are subjected. There is no doubt that an intelligent patient who is animated by a strong desire to get well, and is able to keep himself in subjection, can work wonders, even at home, but there are many patients who are none too intelligent, and there are many more who, when they feel well, cannot be trusted to exercise any restraint upon their inclinations, and taking things altogether there are ample grounds for believing that one of the most important factors in the hygienic treatment of phthisis is constant and intelligent supervision. Dr. Thurnam says: "We expect a patient on coming to us to give up his will and inclination for the time being into our hands, and to allow us to have the direction of even the most simple and apparently trivial details of his daily life."

Taking the cases altogether, the results which have been attained in the British Islands have been good, and in some cases brilliant; but what above all things is important in the papers referred to is the demonstration of the possibility of carrying out the open-air treatment of phthisis in the English climate—cold, foggy, and variable as it too often is in the winter months. On this point the experience of Dr. Philip, of Edinburgh, is most valuable. He gives in a table the actual number of hours spent *outside* by each patient on each day from February

1st to April 30th, 1899, and he says, "There was not a single day throughout the quarter—as there has not been a single day throughout the years during which I have conducted such observations—on which the patients were not outside for a considerable period." This is the point—the possibility of the thing. If this is once accepted, the rest follows. Indeed, our reason for referring to the matter at such length is that we know that among a very considerable section, not only of the public but of medical men, the possibility of carrying on the treatment here described in England is still entirely disbelieved in. Only last week one of our contemporaries amused itself—and perhaps its readers—by holding up to ridicule the attempt made by one one of the hospitals for consumption in London to treat its patients in the open.

